# Hypoxia-induced TMTC3 expression in esophageal squamous cell carcinoma potentiates tumor angiogenesis through Rho GTPase/STAT3/VEGFA pathway

**DOI:** 10.1186/s13046-023-02821-y

**Published:** 2023-09-26

**Authors:** Hongyu Yuan, Zitong Zhao, Jing Xu, Ruiping Zhang, Liying Ma, Jing Han, Weihong Zhao, Mingzhou Guo, Yongmei Song

**Affiliations:** 1 Department of Gastroenterology & Hepatology, The First Medical Center, Chinese PLA General Hospital, 28 Fuxing Road, Beijing, 100853 China; 2 State Key Laboratory of Molecular Oncology, National Cancer Center/National Clinical Research Center for Cancer/Cancer Hospital, Chinese Academy of Medical Sciences and Peking Union Medical College, Beijing, 100021 China; 3 State Key Laboratory of Bioactive Substance and Function of Natural Medicines, Institute of Materia Medica, Chinese Academy of Medical Sciences and Peking Union Medical College, Beijing, 100050 China; 4 Department of Medical Oncology, Hebei Medical University Fourth Affiliated Hospital and Hebei Provincial Tumor Hospital, Shijiazhuang, 050000 Hebei China; 5 Medical Department, Chinese PLA General Hospital, 28 Fuxing Road, Beijing, 100853 China

**Keywords:** Hypoxia, Tumor angiogenesis, TMTC3, IMPDH2, Rho GTPase/STAT3

## Abstract

**Background:**

Hypoxia is one of most typical features in the tumor microenvironment of solid tumor and an inducer of endoplasmic reticulum (ER) stress, and HIF-1α functions as a key transcription factor regulator to promote tumor angiogenesis in the adaptive response to hypoxia. Increasing evidence has suggested that hypoxia plays an important regulatory role of ER homeostasis. We previously identified TMTC3 as an ER stress mediator under nutrient-deficiency condition in esophageal squamous cell carcinoma (ESCC), but the molecular mechanism in hypoxia is still unclear.

**Methods:**

RNA sequencing data of TMTC3 knockdown cells and TCGA database were analyzed to determine the association of TMTC3 and hypoxia. Moreover, ChIP assay and dual-luciferase reporter assay were performed to detect the interaction of HIF-1α and TMTC3 promoter. In vitro and in vivo assays were used to investigate the function of TMTC3 in tumor angiogenesis. The molecular mechanism was determined using co-immunoprecipitation assays, immunofluorescence assays and western blot. The TMTC3 inhibitor was identified by high-throughput screening of FDA-approved drugs. The combination of TMTC3 inhibitor and cisplatin was conducted to confirm the efficiency in vitro and in vivo.

**Results:**

The expression of TMTC3 was remarkably increased under hypoxia and regulated by HIF-1α. Knockdown of TMTC3 inhibited the capability of tumor angiogenesis and ROS production in ESCC. Mechanistically, TMTC3 promoted the production of GTP through interacting with IMPDH2 Bateman domain. The activity of Rho GTPase/STAT3, regulated by cellular GTP levels, decreased in TMTC3 knockdown cells, whereas reversed by IMPDH2 overexpression. Additionally, TMTC3 regulated the expression of VEGFA through Rho GTPase/STAT3 pathway. Allopurinol inhibited the expression of TMTC3 and further reduced the phosphorylation and activation of STAT3 signaling pathway in a dose-dependent manner in ESCC. Additionally, the combination of allopurinol and cisplatin significantly inhibited the cell viability in vitro and tumor growth in vivo, comparing with single drug treatment, respectively.

**Conclusions:**

Collectively, our study clarified the molecular mechanism of TMTC3 in regulating tumor angiogenesis and highlighted the potential therapeutic combination of TMTC3 inhibitor and cisplatin, which proposed a promising strategy for the treatment of ESCC.

**Supplementary Information:**

The online version contains supplementary material available at 10.1186/s13046-023-02821-y.

## Background

Esophageal cancer (EC) is the seventh in terms of incidence and the sixth leading cause of cancer mortality reported by GLOBOCAN 2020 [1]. Esophageal squamous cell carcinoma (ESCC) is the most common histologic subtype of EC in eastern Asia, with relatively low five-year overall survival [2]. Due to occult symptoms in early stage, most of ESCC patients were diagnosed in advanced stage [3]. Yet, there is no effective therapeutic targets such as HER2 in breast cancer. Therefore, it is urgent to explore the molecular mechanism of ESCC carcinogenesis, and identify new targets and therapeutic strategy for therapy.

As tumor grows, oxygen is consumed, and both tumor cells and stromal cells in the tumor microenvironment (TME) are exposed to hypoxia status, in which hypoxia signaling is activated. Hypoxic TME has endowed tumors with abnormal blood vessels and short of blood supply resulting in a more aggressive, treatment-resistant phenotype [4–6]. Hypoxia inducible factor 1 (HIF-1) is thought as a critical transcription factor and orchestrates the cellular response to enhance oxygen consumption and nutrient supply [7]. As an important subunit of HIF-1, the stabilization of HIF-1 alpha (HIF-1α) protein is increased under hypoxic TME, which further directly or indirectly regulated genes involved in cell proliferation, epithelial-to-mesenchymal transition, apoptosis and metastasis or invasion in various tumors [8]. Besides, it is widely accepted that HIF-1α regulated expression of pro-angiogenic factors, such as vascular endothelial growth factor (VEGF), epithelial growth factor (EGF), platelet-derived growth factor (PDGF), transforming growth factor beta (TGF-β), fibroblast growth factor (FGF) [9, 10]. Additionally, HIF-1α functions as a master regulator of oxygen homeostasis and facilitates the generation of reactive oxygen species (ROS) to promote various processes of tumorigenesis, including proliferation, metastasis and angiogenesis [11]. It has been reported that HIF-1α served as an oncogenic transcriptional factor to activate the Wnt/β-catenin pathway in ESCC [12].

Hypoxia is one of inducers of endoplasmic reticulum (ER) stress in the TME to accelerate carcinogenesis [13]. Emerging evidence has revealed that HIF-1α modulates the expression and activity of ER stress sensors under hypoxic conditions. It is proposed that the expression of GRP78 is activated under hypoxic condition to facilitate unfolded protein response (UPR) in the TME [14, 15]. Moreover, hypoxia signaling is shown to activate ER stress especially via PERK and XBP1 [16–18]. For instance, hypoxia activates PERK/eIF2α signaling pathway to protect from oxidative damage in glioblastoma cells [19, 20]. Besides, as a key protein in UPR, the expression of XBP1 is induced in a hypoxic TME to promote tumorigenesis [21]. However, the relevance between ER stress and hypoxia remains unclear in ESCC.

Transmembrane and tetratricopeptide repeat (TPR) repeat-containing protein 3 (TMTC3) is a transmembrane protein located at ER. In our previous study, we demonstrated that TMTC3, as a novel ER stress mediator, enhanced ESCC progression via PERK/ATF4 signaling pathway [22]. Additionally, the RNA-seq of TMTC3 knockdown cells in our previous study indicated that TMTC3 was significantly associated with hypoxia and angiogenesis pathways. However, the function and mechanism of TMTC3 under hypoxia is unclear. In the present study, we not only explored the mechanism of TMTC3 in modulating angiogenesis of ESCC in the hypoxic TME, but also provided an efficient therapeutic strategy against TMTC3 high expression tumors.

## Materials and methods

### Cell lines and cell culture

Ten human ESCC cell lines used in this study were kindly provided by Professor Yutaka Shimada from Kyoto University, Japan. ESCC cell lines, except KYSE150, were maintained in RPMI-1640 with 10% fetal bovine serum (FBS). KYSE150 cell line was cultured in RPMI-1640/Ham’s F12 with 10% FBS. Human umbilical vein endothelial cells (HUVECs) and 293T cells were cultured by DMEM with 10% FBS. All mediums were supplemented with 1% penicillin/streptomycin.

To simulate a hypoxia environment, ESCC cells were cultured in media containing different concentration of cobalt chloride (CoCl_2_) or incubated in a hypoxia oxygen incubator for 24 h, which containing 1% O_2_ and 5% CO_2_.

The untreated cell lines were maintained at 37 °C incubator containing 5% CO_2_. All cell lines were authenticated by short tandem repeat (STR) profiling.

### Reagents and antibodies

The FDA-approved drug library and allopurinol were purchased from Selleck Chemicals. Allopurinol was dissolved with dimethyl sulfoxide (DMSO) for cell experiments. For animal study, Allopurinol was dissolved in 0.5% sodium carboxymethyl cellulose (CMC-Na, purchased from Selleck Chemicals). Cisplatin was purchased from HANSON Pharma and dissolved in 0.9% sodium chloride.

The following antibodies were used in this study: The TMTC3 (sc-398137) and VEGFA (sc-152) antibodies were purchased from Santa Cruz. The β-actin (3700 S) and STAT3 (8768s) antibodies were purchased from Cell Signaling Technology. The HIF-1α (20960-1-AP), IMPDH2 (67663-1-Ig) antibodies were purchased from Proteintech. The p-STAT3 (bs-1658R) antibody was purchased from Bioss. The Flag (F3165) and β-tubulin (T5201) antibodies were purchased from Sigma. The p-JAK2 (ab32101) antibody was purchased from Abcam. The JAK2 (A19629) antibody was purchased from ABclonal Technology.

The siRNAs of IMPDH2 were synthesized by GenePharma (Suzhou, China). The siRNA sequences for IMPDH2 and primers for qPCR were listed in Supplementary Table 1.

### Dual-luciferase reporter assay

The sequence of TMTC3 promoter was subcloned into the firefly luciferase vector. The sequence of HIF-1α was subcloned into pcDNA3.1 vector. The TMTC3 promoter firefly luciferase vector along with Renilla luciferase vector (used as a loading control) were co-transfected with pcDNA3.1-HIF-1α or pcDNA3.1-empty (negative control) vector into 293T or KYSE450 cells. The cells were harvested for 48 h post-transfection and lysed with lysis buffer. The luciferase activity was measured with a Dual-Glo® Luciferase Assay System (Promega, USA).

### Chromatin immunoprecipitation (ChIP) assay

ChIP assay was performed by Pierce Magnetic ChIP Kit (Thermo Fisher Scientific) following the manufacturer’s instructions. The antibody against HIF-1α was incubated with cross-linked chromatin of ESCC cells followed by addition of magnetic beads. qPCR was conducted to detect the promoter of TMTC3 using purifying the coprecipitated DNA. The primers were listed in Supplementary Table 1.

### Co-immunoprecipitation assay

The cells were scraped and collected by centrifugation at 3,000 rpm for 5 min. The cell lysate and immunoprecipitation were performed by using appropriate antibodies, TMTC3 or IMPDH2. After incubated with the antibodies at 4℃ for 12 h, the protein A/G beads were added and incubated for another 6 h. Immunoprecipitants were collected by centrifugation at 3,000 rpm for 3 min, washed five times with IP buffer, and eluted with elution buffer and neutralized with neutralization buffer, and then heated at 100℃ for 10 min in equal volume loading buffer for SDS-PAGE.

### Conditioned medium collection

The KYSE410 or KYSE450 cells were seeded into 6-well plate at a density of 2 × 10^5^ cells/well and cultured overnight at 37℃ incubator. Then, the cells were transfected with siRNA of TMTC3 or negative control.

For HUVECs tube formation assay, the serum-free medium was replaced after 24 h. Then, cell culture supernatants were collected at 48 h and centrifuged at 2,000 rpm to remove cell debris.

For HUVECs wound healing assay and Transwell HUVECs migration assay, cell culture supernatants were collected at 48 h and centrifuged at 2,000 rpm to remove cell debris.

### Transwell HUVECs migration assay

A transwell system was used to estimate the migration ability of HUVECs affected by ESCC cells with or without TMTC3 in vitro. ESCC cells transfected with siRNA of TMTC3 or negative control and condition media were collected. The condition media were added into the lower chamber of transwell and HUVECs (5 × 10^4^) were seeded on upper chamber. After incubation for 3 h at 37℃, the HUVECs in lower chamber were fixed and stained with crystal violet. The number of cells were counted in each field by Image J software.

### HUVECs tube formation assay

The condition media were collected and stored at -80℃. The 24-well plate was precoated with 50% Matrigel and HUVECs (5 × 10^4^) suspended in condition medium were seeded upon Matrigel. Tube formation was observed after incubation for 3 h at 37℃. The number of tubular structures was counted in each field by Image J software [23].

### HUVECs wound healing assay

Condition media were collected and stored at -80℃. The HUVECs cells (3 × 10^5^) were seeded on 6-well plate. 100% confluent cells were scratched by the pipette tips and cultured by condition media. The open area was captured and assessed by Image J software after 18 h later.

### Cell proliferation assays

The cell proliferation was detected by Real Time Cell Analyzer (xCelligence, RTCA, USA) as described previously [24]. In brief, the indicated cells were added into 96-well plate. The impedance signals were recorded every 15 min. After 24 h, the cells were treated with different concentrations of allopurinol or CDDP, combination. The plate was placed on the machine and detected with impedance changes, which are positively associated with the number of adherent cells. Cell index was normalized at the final detection time point before treatment.

### Chicken embryo chorioallantoic membrane (CAM) assay

CAM assay were performed with seven day-old chicken embryos (Vital River Co., Beijing, China) as described previously [23]. In briefly, a window about 1.0 cm in diameter was opened in the eggshell to expose the CAM. Conditioned medium (200 µl) was added to incubate the CAM for 3–5 days. CAMs were fixed and photographed and the effect of conditioned medium on angiogenesis was measured in each field.

### Immunohistochemical staining and scoring

IHC staining was performed as previously described [25]. The ESCC tissue microarray (D193Es01, Bioaitech Co.,Ltd., Xi’an, China) was used in this assay. Immunostaining score was calculated based on the percentage of positive cells and the intensity score of the staining. The percentage of positive cells was determined as follows: 0–4% positive cells, score of 0; 5–25% positive cells, score of 1; 26–50% positive cells, score of 2; 51–75% positive cells, score of 3; 76–100% positive cells, score of 4. The intensity score of the staining was determined as follows: 0 = negative, 1 = weakly positive, 2 = moderately positive, 3 = strongly positive. The final immunoreactive score was determined by multiplying the positive intensity and the positive extent scores, yielding a range from 0 to 12.

### Rho GTPase activity detection

Levels of GTP-bound Rac1, GTP-bound CDC42 and GTP-bound RhoA were detected using an RhoA/Rac1/Cdc42 Activation Assay Combo Kit (Cell Biolabs, #STA-405) according to the manufacturer’s recommendations. In briefly, indicated cells were starved in serum-free medium for 24 h and stimulated with 10% FBS for 2 h, then collected by lysis buffer. Cell lysates were incubated with Rhotekin RBD or PAK1 PBD agarose beads for 1 h at 4 ℃. Immunoblotting was used to detect GTP-RhoA, GTP-RAC1 and GTP-CDC42.

### High throughput screening for TMTC3 inhibitor

The FDA-approved drug library was purchased from Selleck (Catalog No. L1300). KYSE450 cells were seeded in 6-well plates and incubated overnight at 37℃ incubator. When the cells reached at 50% confluence, each drug was added to individual wells. Plates were incubated for 48 h. Total RNA from samples was extracted using TRnaZol RNA Kit (NCM Biotech, Suzhou, China) and converted to cDNA. The expression of TMTC3 was measured by Real-Time PCR and normalized by β-actin. The fold change of TMTC3 expression was calculated compared to parental cells. The primer sequences were listed in supplementary Table 1. The result of screening data was provided in supplementary Table 2.

### Tumor xenografts in nude mice

Four-week-old male BALB/c nude mice were purchased from Beijing HFK Bioscience Co.,LTD (Beijing, China). KYSE450 cells were subcutaneously inoculated into back of the mice (2 × 10^6^ cells in 100 µl PBS). When tumor volume grew to 5 × 5 × 5 mm^3^, the mice were divided into four groups randomly, including placebo, allopurinol treatment, cisplatin treatment and combination treatment. Allopurinol was given by gavage administration at the dosage of 100 mg/kg three times a week, and cisplatin was given by intraperitoneal injection at the dosage of 2 mg/kg once a week. The length (L) and width (W) of the tumor were measured using calipers every 3 days for 3 weeks. Tumor volume was calculated using the following equation: volume (mm^3^) = L × W^2^ × 0.5.

### ROS accumulation analysis

The ROS levels were determined using ROS assay kits (Beyotime biotechnology, Shanghai, China). Conditioned cells were cultured overnight in 6-well plate. After resuspension in medium without FBS, DCFH-DA (dilution ratio 1:2000) was added, and the cells were stained at 37℃ for 20 min in dark. The cells were washed twice with PBS, and the levels of ROS were evaluated by flow cytometry at 488 nm.

### Statistical analysis

Each experiment was performed in triplicate, and all data are expressed as the mean ± standard deviation. Unpaired Student’s t-test was used for biological experiment analysis. One-way analysis of variance (ANOVA) for multiple-group analysis was conducted. P < 0.05 was considered to indicate statistical significance. All statistical analyses were performed with GraphPad Prism 8 (GraphPad Software Inc.) or SPSS 22 (SPSS Inc.).

## Results

### Ectopic expression of TMTC3 under hypoxic condition

**Fig. 1:**
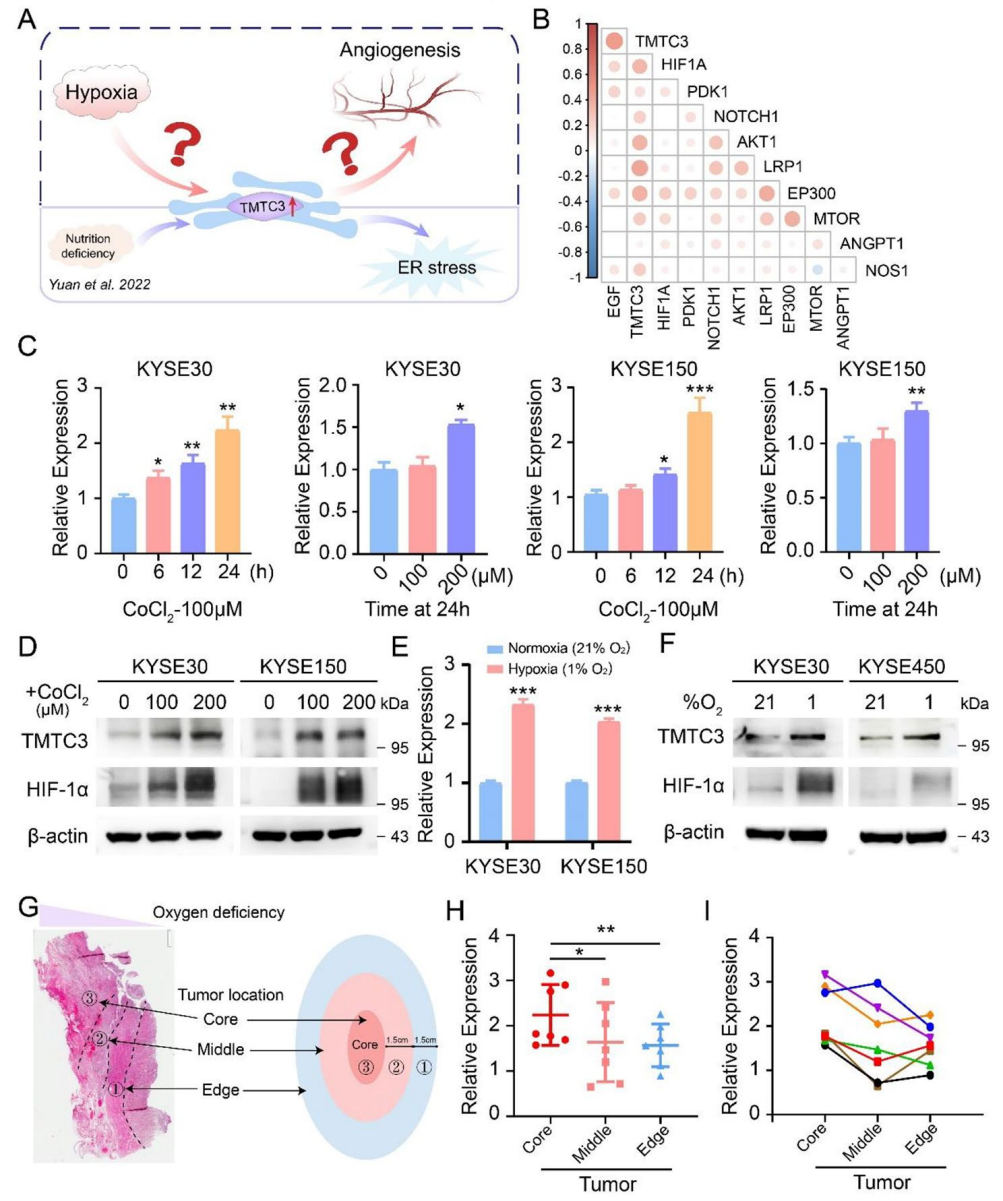
Increased expression of TMTC3 under hypoxic conditions. (**A**) Schematic diagram depicting the aim of this study. (**B**) Cross correlation analysis of TMTC3 expression and genes in HIF-1α pathway from TCGA database. **C** and **D**. ESCC cells were treated by CoCl_2_ with different concentration for 24 h or 100 µM CoCl_2_ for different times. The TMTC3 mRNA levels (**C**) and protein levels (**D**) were detected. **E** and **F**. ESCC cells were cultured in a hypoxia oxygen incubator for 24 h, and mRNA expression (**E**) and protein expression (**F**) were analyzed. **G**. Schematic diagram depicting the tumor locations of ESCC. **H**. The expression of TMTC3 at different tumor location in ESCC (n = 7). **I**. The expression of TMTC3 in each group tissue of ESCC. Each color represents a group of samples from the same patient (n = 7). All data are expressed as the mean ± SD. *, p < 0.05; **, p < 0.01; ***, p < 0.001. n = 3

In our previous study, the expression of TMTC3 was upregulated under ER stress condition [22]. Hypoxia as an another ER stress inducer, could promote tumor angiogenesis. We next investigated whether TMTC3 was regulated under hypoxic microenvironment to induce the formation of blood vessels (Fig. 1A). The strong correlation between TMTC3 and genes in hypoxia signaling pathway from TCGA database was validated (Fig. 1B). Additionally, RNA-seq analysis in TMTC3 knockdown cells revealed that TMTC3 is closely related to hypoxia signaling pathway and angiogenesis pathway (Fig. S1A). Hypoxic microenvironment was a key factor for inducing ER stress in tumor, therefore we supposed aberrant expression of TMTC3 might be modulated under hypoxia. To further validate the hypothesis, we examined the expression of TMTC3 upon hypoxia in ESCC cells. The addition of CoCl_2_ increased both mRNA and protein levels of TMTC3 dependent on doses in ESCC cells (Fig. 1C and D). Additionally, ESCC cells were cultured in a hypoxia oxygen incubator for 24 h and analyzed TMTC3 expression at RNA and protein levels, which indicated obviously upregulated expression (Fig. 1E and F). Moreover, TMTC3 was expressed at higher levels in the central part of the tumor compared with the leading edge of ESCC tumor tissues (Fig. 1G, H and I). These results suggested the elevated expression of TMTC3 under hypoxic TME.

### TMTC3 regulated by HIF-1α promoted HUVECs angiogenesis

**Fig. 2:**
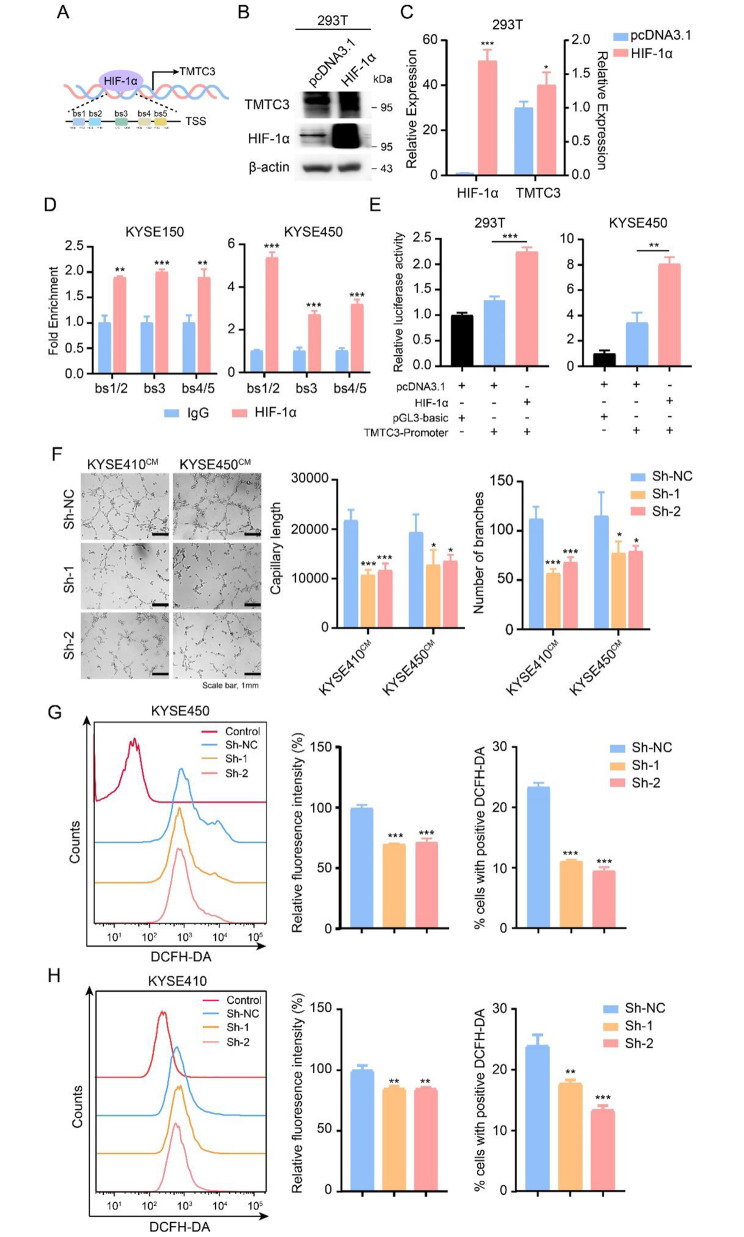
TMTC3 was regulated by HIF-1α and modulated the angiogenesis of HUVECs. **A**. Schematic diagram depicting the positions of the binding sites of HIF-1α in the TMTC3 promoter from the JASPAR database. TSS, transcription start site. **B** and **C**. The levels of TMTC3 was increased both in protein (**B**) and RNA level (**C**) after transfected with HIF-1α. **D**. Quantification of ChIP analysis of the interaction between HIF-1α and TMTC3 promoter. IgG was used as a negative control. **E**. Dual-luciferase reporter assay demonstrating the effects on TMTC3 transcription activity in the indicated cells following HIF-1α overexpression. **F**. Conditioned medium from TMTC3 knockdown cells decreased HUVECs capillary tube formation. The capillary length and number of branches were counted by Image J software. **G** and **H**. ROS accumulation was analyzed by flow cytometry with DCFH-DA probe staining in KYSE450 (**G**) and KYSE410 (**H**) cells. The fluorescence intensity and positive cells in each group were calculated. All data are expressed as the mean ± SD. *, p < 0.05; **, p < 0.01; ***, p < 0.001. n = 3

HIF-1α is a critical transcription factor induced under hypoxia condition and regulates the expression of genes involved in angiogenesis, proliferation, invasion and metastasis in various tumors. Therefore, we used JASPAR database to predict binding sites of HIF-1α in TMTC3 promoter (Fig. 2A). Moreover, overexpression of HIF-1α significantly increased TMTC3 level in 293T cells (Fig. 2B and C). To further verify the modulated relationships between HIF-1α and TMTC3, ChIP-qPCR and dual-luciferase reporter assays were performed. As shown in Fig. 2D, HIF-1α was enriched in the region predicted by JASPAR database from the transcription start site of the TMTC3 promoter. Furthermore, the sequence of TMTC3 promoter was cloned into a firefly luciferase reporter vector, which showed that luciferase activity was obviously enhanced in 293T cells and KYSE450 cells that were co-transfected with HIF-1α and TMTC3 promoter compared with another two control groups (Fig. 2E). The strong correlation between HIF-1α and TMTC3 was further observed in EC through GEPIA2 and TIMER2.0 databases (Fig. S1B and S1C).

Next, the effect of TMTC3 on the angiogenesis of human umbilical vein endothelial cells (HUVECs) was detected by conducting tube formation experiment. As shown in Fig. 2F, the ability of tube formation was significantly inhibited in the TMTC3 knockdown cells in comparing with control group. Wound healing assay showed that HUVECs, which cultured with conditioned media of TMTC3 knockdown cells, had less migration abilities than those of the control group (Fig. S1D). Also, cellular ROS is associated with tumor angiogenesis process under hypoxia condition [26]. To further explore the effects of TMTC3 on ROS production in ESCC cells, flow cytometry assays were performed. As shown in Fig. 2G and H, the results revealed that cellular ROS was obviously decreased in TMTC3 knockdown cells. Thus, these data strongly suggested that the expression of TMTC3 was regulated by HIF-1α, a critical transcription factor under hypoxia, and involved in tumor angiogenesis in ESCC.

### Identification of an interacting protein with TMTC3

**Fig. 3:**
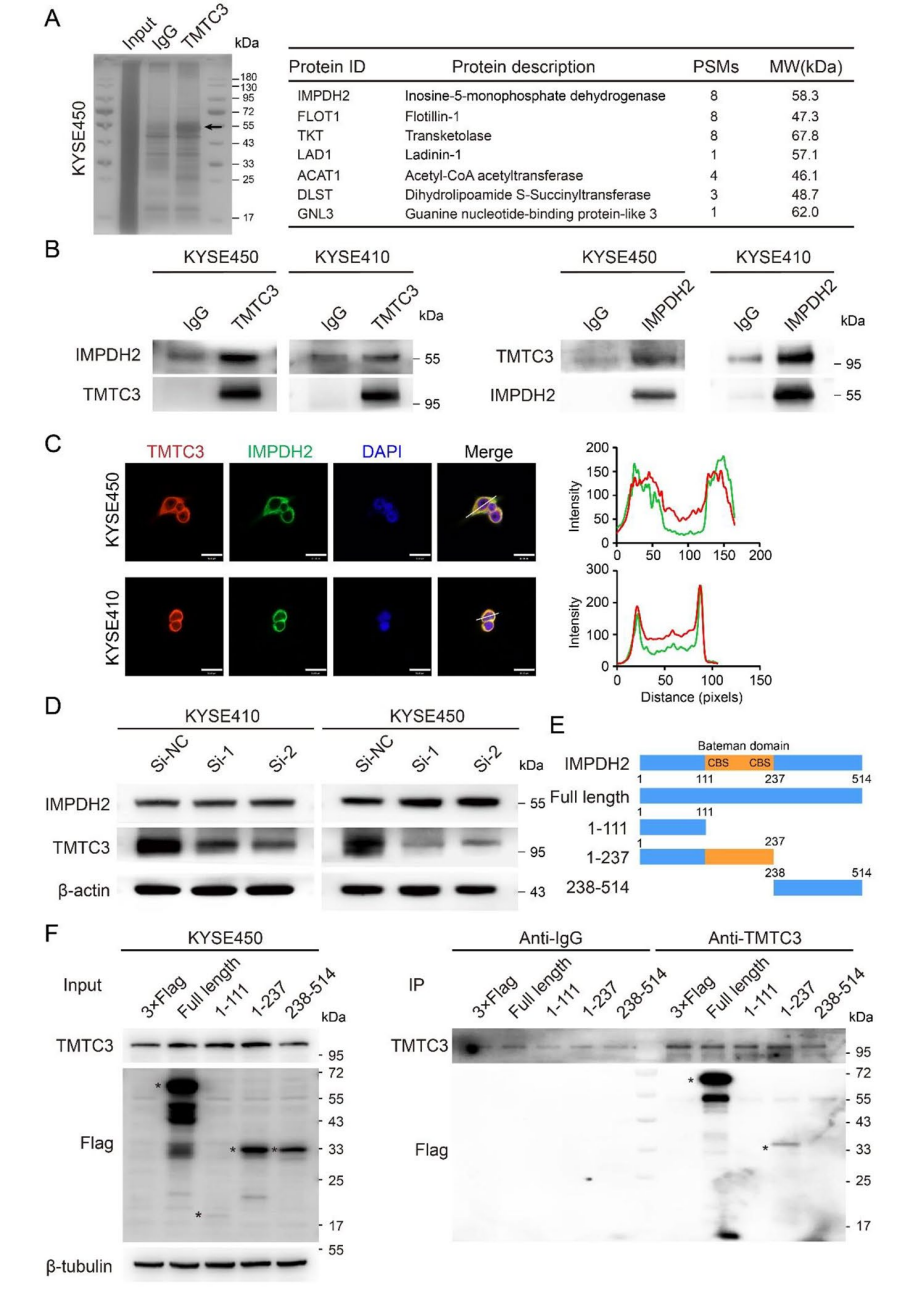
Identifying the interaction between TMTC3 and the Bateman domain of IMPDH2. (**A**) Proteins that interacted with TMTC3 were detected by silver staining SDS-PAGE gel and mass spectrometry. The representative proteins in the differential band around 55 kDa were analyzed and listed. (**B**) The interaction between TMTC3 and IMPDH2 was validated by co-immunoprecipitation assays with anti-TMTC3 or anti-IMPDH2 antibody or IgG, respectively. (**C**) Immunofluorescence assay was performed on KYSE450 and KYSE410 cells with the antibodies indicated on the top. Plots of pixel intensity along the white line from the images, colors as in merged images. Scale bars, 30 μm. (**D**) The effect on IMPDH2 expression in TMTC3 knockdown cells was detected by western blot assay. (**E**) Schematic representation of IMPDH2 truncated mutants. (**F**) Indicated Flag-tagged IMPDH2 mutants were transfected into KYSE450 cells, followed by immunoprecipitation with anti-TMTC3 antibody and immunoblotting with anti-Flag antibody

We wondered what the molecular mechanism underlying TMTC3 for the angiogenesis in ESCC might be. Co-immunoprecipitation assay using TMTC3 antibody was conducted in KYSE450 cells and the differential band between IgG and TMTC3 group around 55 kDa was selected for further analysis by mass spectrometry (Fig. 3A). We noticed that inosine-5’-monophosphate dehydrogenase 2 (IMPDH2), a rate-limiting enzyme in de novo GTP biosynthesis and plays critical roles in malignant progression of tumors [27], was detected by TMTC3 specific antibody compared with negative control (Fig. 3A). To investigate the functional role of IMPDH2 in ESCC, we induced silencing of IMPDH2 by siRNA in ESCC cells. IMPDH2 was significantly knocked down at both protein and mRNA levels in three ESCC cell lines (Fig. S2A and S2B). Subsequent cell proliferation and transwell assays indicated that knockdown IMPDH2 obviously suppressed the abilities of cell proliferation, invasion and migration in KYSE410 and KYSE450 cells (Fig. S2C and S2D).

Next, the interaction between TMTC3 and IMPDH2 was further confirmed by co-immunoprecipitation assay with the lysates of KYSE410 and KYSE450 cells (Fig. 3B). Besides, immunofluorescence analysis was also performed and the results showed that TMTC3 colocalized with IMPDH2 in the cytoplasm (Fig. 3C). However, knockdown TMTC3 did not affect IMPDH2 expression in cells (Fig. 3D). Further analysis of mass spectrometry data found that the peptides detected in lysates was located at the Bateman domain of IMPDH2, which is required for their allosteric regulation. To further validate the IMPDH2 domain(s) that mediated interaction with TMTC3, we generated Flag-tagged full-length or truncated IMPDH2 plasmids (Fig. 3E). Co-immunoprecipitation assay was conducted by TMTC3 antibody using protein A/G agarose beads in KYSE450 and KYSE180 cells transfected with the established IMPDH2 plasmids (Fig. 3F and Fig. S2E). The immunoblotting analysis demonstrated that the region of IMPDH2 containing CBS motif (111-237aa) (Fig. 3E) was required for TMTC3-IMPDH2 interactions. These results indicated that TMTC3 may bind to the Bateman domain of IMPDH2 and regulate its enzyme activity.

### Knockdown TMTC3 decreased HUVECs angiogenesis and reversed by IMPDH2

**Fig. 4:**
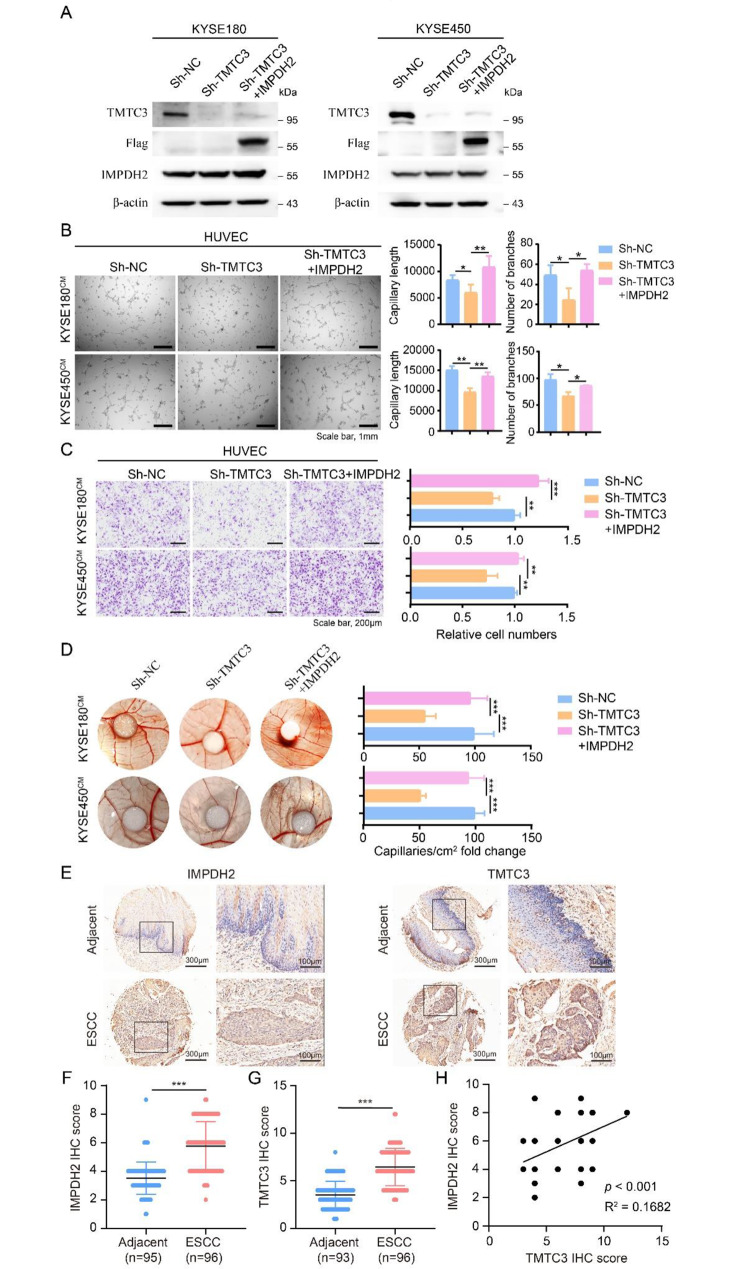
IMPDH2 overexpression relieved the angiogenesis inhibition caused by TMTC3 knockdown. (**A**) The overexpression efficiency of IMPDH2 in TMTC3 knockdown cells. (**B**) Representative micrographs of the HUVECs tube formation treated with indicated condition medium (left). Quantification of the capillary length and number of branches (right). (**C**) The migration ability of HUVECs was measured by transwell assay as described in Materials and Methods. The number of migrated cells were counted by Image J software. (**D**) Representative images of the blood vessels formed in the CAM assay, after incubation with conditioned medium from the indicated cells. The results are summarized in the graphs as vessel density. (**E**) Representative IHC images for IMPDH2 and TMTC3 in ESCC tissue array. **F** and **G**. IHC staining score for IMPDH2 (**F**) and TMTC3 (**G**). **H**. Correlation of IMPDH2 IHC score and TMTC3 IHC score. All data are expressed as the mean ± SD. *, p < 0.05; **, p < 0.01; ***, p < 0.001. n = 3

Furthermore, we wondered whether TMTC3 regulated the angiogenesis of HUVECs via IMPDH2. The IMPDH2 plasmid was transfected into TMTC3 knockdown cells and the overexpression efficiency of IMPDH2 was detected by western blot in KYSE180 and KYSE450 cells (Fig. 4A). The tube formation of HUVECs was significantly impaired by IMPDH2 overexpression in the TMTC3 knockdown cells (Fig. 4B). Additionally, the migration ability of HUVECs was reversed by upregulated IMPDH2 compared with TMTC3 inhibition cells through transwell assay (Fig. 4C) and wound closure assay (Fig. S3A and S3B). To monitor the effect of IMPDH2 on TMTC3 induced angiogenesis in vivo, the vascular formation in chick chorioallantoic membrane (CAM) assay was supported by IMPDH2 overexpression in comparison with TMTC3 downregulation (Fig. 4D). Besides, IMPDH2 rescued ROS production induced by TMTC3 silencing (Fig. S3C and S3D). These data suggested that IMPDH2 was essential for TMTC3-mediated angiogenesis in ESCC.

To further investigate the significance of IMPDH2 and TMTC3 in ESCC, an IHC assay for evaluating the protein levels of IMPDH2 and TMTC3 were performed via tissue microarrays. The results indicated the expression levels of both of IMPDH2 and TMTC3 were significantly upregulated in ESCC tissues compared with adjacent peritumoral tissues (Fig. 4E, F and G). Additionally, the protein level between IMPDH2 and TMTC3 existed weak positive correlation (Fig. 4H). These results suggested the levels of TMTC3 and IMPDH2 were both upregulated in ESCC.

### Downregulation of TMTC3 inhibited STAT3 signaling pathway through Rho GTPase

**Fig. 5:**
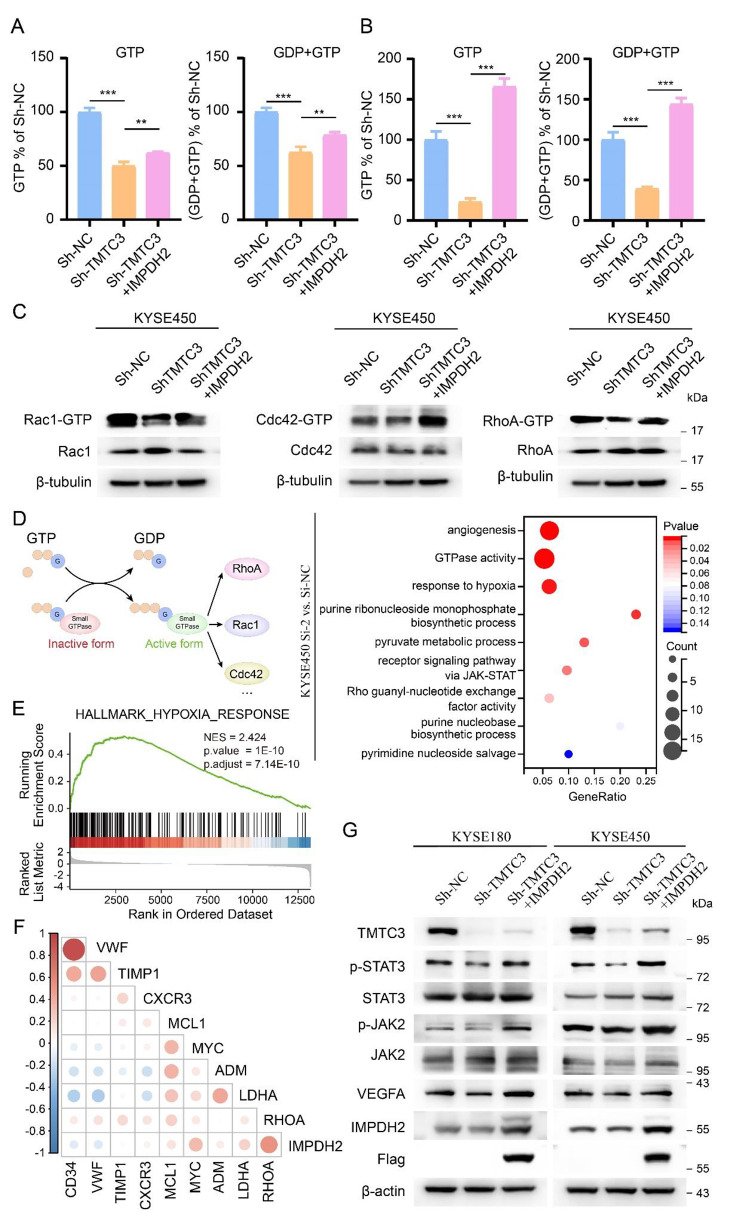
TMTC3 induced the production of GTP and activity of Rho GTPase through STAT3 pathway. **A** and **B**. The GTP and GDP levels in TMTC3 knockdown cells or IMPDH2 overexpression cells were detected by mass spectrometry in KYSE180 (**A**) and KYSE450 (**B**) cells, respectively. **C**. Blot analyses for GTP-bound RhoA, Rac1, and Cdc42 levels in TMTC3 knockdown cells, with or without overexpression of IMPDH2, following Rho GTPase pull-down experiments. Total protein levels were detected from whole cell lysate. **D**. Schematic representation of the GTPase cycle between their GDP-bound and GTP-bound conformations (left). The enrichment terms of differential expressed genes in IMPDH2 knockdown cells (right). **E**. Gene set enrichment analysis (GSEA) of RNA-seq data on IMPDH2 knockdown cells. NES, normalized enrichment score. **F**. Cross correlation analysis of IMPDH2 expression and genes in HIF-1α pathway from TCGA database. **G**. Blot analyses for the downstream proteins of TMTC3 in TMTC3 knockdown cells, with or without overexpression of IMPDH2. All data are expressed as the mean ± SD. **, p < 0.01; ***, p < 0.001. n = 3

GTP is particular important for protein synthesis and required in rapidly dividing tumor cells, and its biosynthesis is manipulated by IMPDH2. Additionally, the binding domain of IMPDH2 with TMTC3 was Bateman domain, which is required for allosterically modulating the catalytic activity [28]. Thus, we investigated intracellular GTP levels in TMTC3 knockdown cells using mass spectrometry. The results showed silencing TMTC3 could obviously decreased intracellular GTP levels (Fig. S3E), further rescued by IMPDH2 overexpression both in KYSE180 and KYSE450 cells, respectively (Fig. 5A and B). The changes in intracellular GTP have been validated important for the activity of GTPase and cell invasion [29]. Among various types of cancer especially ESCC, the activity of Rho GTPase, in particular those of the family (including most studied members RhoA, Rac1, and Cdc42), have been strongly associated with tumor progression [30]. Thus, we explored whether TMTC3 possessed intrinsic ability to regulate activity of the above Rho GTPase through IMPDH2. As expected, silencing TMTC3 cells demonstrated lower amounts of the corresponding GTP-bound RhoA, GTP-bound Rac1 and GTP-bound CDC42, whereas the amounts of the above activity GTPase were rescued by IMPDH2 (Fig. 5C).

Based on the above results, we were interested in identifying signaling pathway affected by TMTC3-IMPDH2 axis in regulating activity of GTPase. Transcriptome profiling of silencing IMPDH2 cells and control cells indicated that IMPDH2 was associated with STAT3 signaling pathway, hypoxia response and angiogenesis process by GO and gene set enrichment analysis (GSEA) (Fig. 5D and E). Additionally, the strong correlation between IMPDH2 and genes in hypoxia pathway was further validated in ESCC in TCGA database (Fig. 5F). Among these pathways, STAT3 signaling pathway could be activated by Rho GTPase via induction of phosphorylation of endogenous STAT3 [31–33]. Additionally, STAT3 induces tumor angiogenesis via elevating VEGFA levels in various tumors [34, 35]. Thus, to further reveal whether TMTC3 regulated STAT3 pathway through IMPDH2, we examined its regulation on phosphorylation of STAT3 in TMTC3 silencing cells and IMPDH2 overexpression cells. The rescued assay of western blot showed that the decrease of phosphorylation of STAT3 and VEGFA induced by silencing TMTC3 was reversed after transducing with IMPDH2 in both KYSE180 and KYSE450 cells (Fig. 5G). The above results confirmed that TMTC3-IMPDH2 axis affected STAT3 pathway by regulating the activity of Rho GTPase.

### Screening of allopurinol as a potent and selective TMTC3 inhibitor

**Fig. 6:**
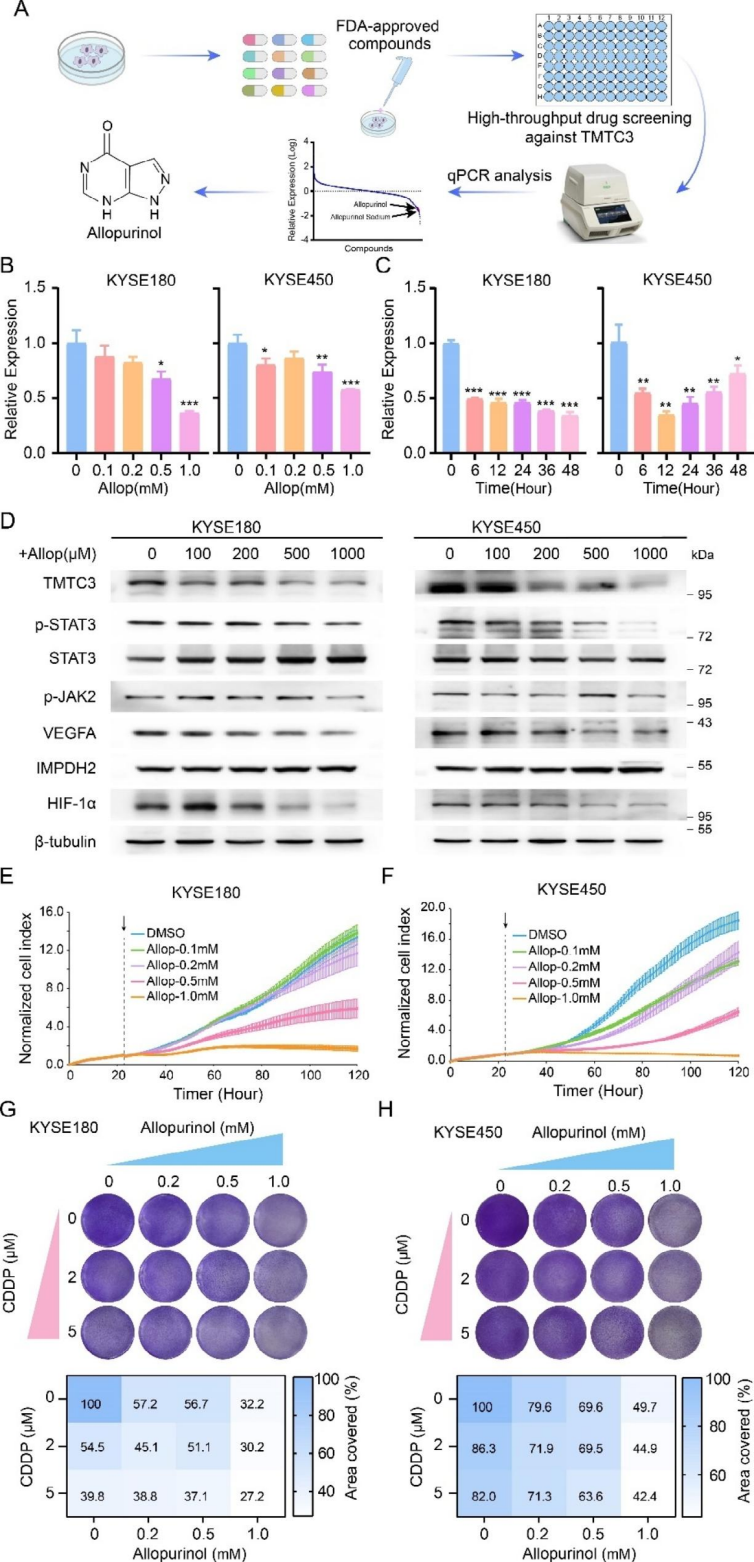
A TMTC3 inhibitor was identified and verified to enhance the efficacy of cisplatin. **A**. A high-throughput drug screening strategy against TMTC3. **B** and **C**. The mRNA expression of TMTC3 in ESCC cells treated by allopurinol in different doses (**B**) or different periods (**C**). **D**. The protein levels of TMTC3 and its downstream pathway treated with different doses of allopurinol. **E** and **F**. The ability of cell proliferation treated with different doses of allopurinol in KYSE180 (**E**) and KYSE450 (**F**) cells. **G** and **H**. Synergistic response to the combination of allopurinol and cisplatin in KYSE180 (**G**) and KYSE450 (**H**) cells (up). The percentage of clonogenic formation was calculated (down). All data are expressed as the mean ± SD. *, p < 0.05; **, p < 0.01; ***, p < 0.001. n = 3

To further screening inhibitor of TMTC3, we performed high throughput screening using a library of 1953 compounds that FDA approved (Fig. 6A, Fig. S4A and supplementary Table 2). Among these, allopurinol is an inhibitor of xanthine oxidase, and used in the treatment of hyperuricemia and gout for over 50 years [36]. In addition, it has been reported that allopurinol decreases the expression of HIF-1α protein [37, 38] and regulated STAT3 signaling pathway [39]. Thus, we further validated the effect of allopurinol on the expression of TMTC3. The results showed that the expression of TMTC3 at mRNA level were decreased significantly by allopurinol in a dose and time-dependent manner in KYSE180 and KYSE450 cells (Fig. 6B and C). In line with the previous results, allopurinol also reduced the protein levels of TMTC3 and HIF-1α protein, and also inhibited STAT3 signaling pathway and VEGFA levels (Fig. 6D). Additionally, treatment with allopurinol significantly reduced proliferation ability of KYSE180 and KYSE450 cells (Fig. 6E and F). The cell counting kit-8 (CCK8) cytotoxicity assay indicated that knockdown TMTC3 increased the half-maximal inhibitory concentration (IC50) of allopurinol (Fig. S4B), suggesting the relevance between TMTC3 levels and allopurinol. Additionally, the intracellular GTP levels were decreased by allopurinol in a dose-dependent manner (Fig. S4C and S4D), consisting with the results in silencing TMTC3 cells. Taken together, these data suggested that allopurinol obviously inhibited the expression of TMTC3, especially in cells with high TMTC3 expression.

### Allopurinol conferred the sensitivity of CDDP in vitro and in vivo

Platinum-based adjuvant and neoadjuvant chemotherapy is the standard treatment for ESCC patients, but its resistance is a major cause of treatment failure. Based on the above findings and the fact that allopurinol can effectively inhibited cell proliferation, we hypothesized that allopurinol might improve treatment efficiency of ESCC. The inhibition of cell proliferation was significantly more effective with the combination of allopurinol and cisplatin in both KYSE450 and KYSE180 cells (Fig. S4E). The result of clonogenic assay also indicated that combination treatment showed synergistic effect in ESCC cells (Fig. 6G and H).

**Fig. 7:**
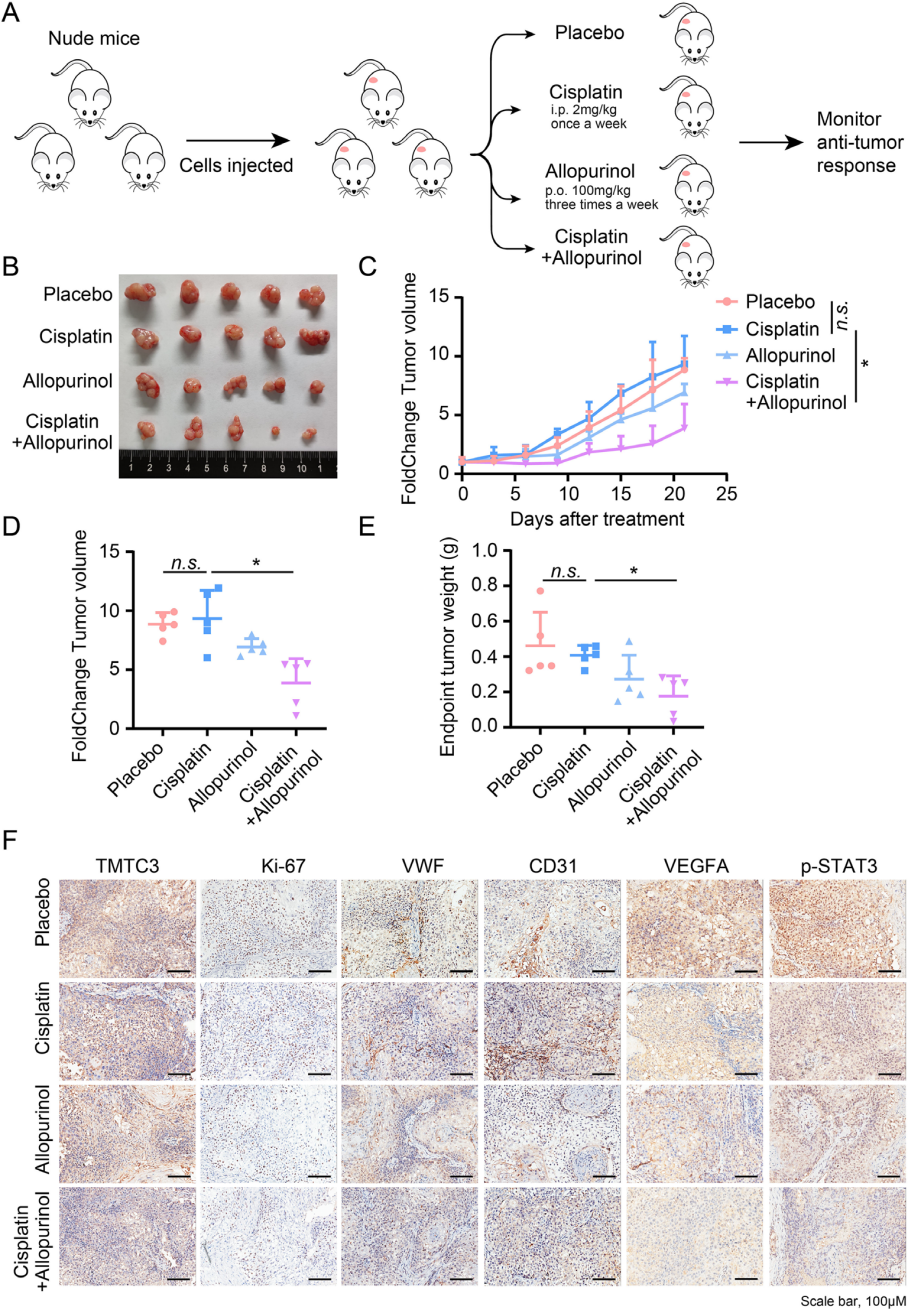
Effects of allopurinol and cisplatin on tumor growth in KYSE450 xenograft model. (**A**) Schematic of mouse studies. (**B**) Representative tumor images of each group at the end of treatment. (**C**) Curves of percent change in tumor volume for each group. (**D**) Dot plot of percent change in tumor volume at the end of the experiment. (**E**) Dot plot of tumor weight at the end of the experiment. (**F**) Representative images of TMTC3, Ki67, VWF, CD31 and p-STAT3 staining in KYSE450 xenografts. All data are expressed as the mean ± SD. *n.s*., no significance; *, p < 0.05; **, p < 0.01. n = 5

In order to better achieve clinical transformation, the effect of combination therapy was detected by in vivo experiment. KYSE450 cells were implanted subcutaneously into mice and treated with either vehicle, cisplatin, or allopurinol for TMTC3 inhibitor alone, or in combination (Fig. 7A). We observed significantly impaired tumor volume in mice treated with cisplatin and allopurinol combination therapy compared with cisplatin single drug therapy (Fig. 7B, C and D and Fig. S4F), while mice weights in combination treatment group were slightly loss (Fig. S4G). In line with tumor volumes, the weights of tumors at the end of the experiment were obviously decreased in combination therapy in comparison to those of other groups (Fig. 7E). Immunohistochemistry analysis revealed lower expression of TMTC3, a decrease in the proliferation maker (Ki-67), micro vessel density (VWF, CD31 and VEGFA), and phosphorylation of STAT3 after combination treatment (Fig. 7F). Taken together, these data revealed allopurinol enhanced the sensitivity of cisplatin in ESCC xenograft.

## Discussion

Hypoxia is one of most typical features in the TME of solid tumor, and HIF-1signaling pathway is activated in tumor cells to adapt to hypoxic TME [40]. Increasing evidence has suggested that hypoxia plays an important regulatory role of ER homeostasis [13, 41, 42]. Recent studies showed that HIF-1α was a co-regulator of XBP1 and induced its expression in triple negative breast cancer [18, 21]. Additionally, hypoxic exposure results in the activation of oxygen-dependent ER-localized oxidoreductase 1 alpha (ERO1α) that could promote ER stress [43, 44]. However, the relationship between ER stress and hypoxia remains unclear in ESCC. In our previous study, we identified an ER stress mediator TMTC3, which regulated ER stress process [22]. Furthermore, in the present study, we determined that high TMTC3 expression in ESCC may be caused by hypoxia and regulated by HIF-1α, a critical transcriptional regulator of hypoxia, which established a link between ER stress and hypoxia in ESCC. The ROS generation is elevated triggered by hypoxia and increases the stabilization and activation of HIF-1α in various tumor cells [45]. Accumulating evidence indicates the production of ROS could stimulate angiogenic or neovascularized response [46]. Our study confirmed that knockdown TMTC3 obviously inhibited ROS generation and angiogenesis formation.

In current study, our results identified that IMPDH2 interacted with TMTC3 by co-immunoprecipitation and Mass Spectrometry. IMPDH2 is known as a key rate-limiting enzyme in the de novo synthesis of GTP, which catalyzes the oxidation from IMP to XMP [27]. The Bateman domain composed of pairs of cystathionine β-synthase (CBS) motifs acts as allosteric modulator in sensing the cellular energy status and regulating enzymatic activity [47, 48]. Interestingly, by analyzing Mass-spectrometry peptides, we suggested that the interaction domain of IMPDH2 with TMTC3 was Bateman domain in this study, and further experiments validated this suppose using truncated plasmids of IMPDH2. Our further experiments showed that the level of intracellular GTP in TMTC3 knockdown cells was lower compared with control group, but reversed by IMPDH2 overexpression. Intracellular GTP concentration is elevated in several types of tumors compared that in normal cells [49, 50]. This gives an advantage for GTP and enhances the ratio of active GTP-bound small G protein (including Rho GTPase), causing the persistent activation [51]. Despite this, it has been reported that IMPDH2 increased RAC1 activity via direct interaction [29]. Here we reported that the Rho GTPase activity, including RhoA, RAC1 and Cdc42, was suppressed in TMTC3 knockdown cells, but reversed by IMPDH2 overexpression.

In this study, RNA-seq analysis in IMPDH2-knockdown cells indicated that the IMPDH2 expression was associated with STAT3 signaling pathway. Previous studies showed that the Rho GTPase was able to induce STAT3 activation [32, 52]. In colorectal cancer, expression of RhoA is elevated and further results in induction of Tyr705 phosphorylation of STAT3 [53]. As with colorectal cancer, chemical RhoA inhibitor attenuated STAT3 activation in hepatoma cell line [54]. Besides, the active form of Rac1 promotes invasion of colorectal cancer cells through activation of STAT3 pathway [55]. The inhibitor of Rac/Cdc42 significantly decreased the activity of STAT3 in metastatic cancer [56]. Thus, we suggested that TMTC3 could mediate the activation of STAT3 signaling pathway through IMPDH2, which was further validated by our results. Additionally, the activation of STAT3 promotes the expression of VEGFA and further induces angiogenesis in various tumors [34, 35]. This conclusion also provided evidence that TMTC3 facilitated tumor angiogenesis through STAT3 signaling pathway in ESCC.

Platinum-based chemotherapy is used as a common regiment for patients with advanced ESCC. The chemoresistance of platinum is a major obstacle that affects its therapeutic efficacy. In the present study, we found that a inhibitor of TMTC3, allopurinol, could improve chemotherapy efficacy of cisplatin. Allopurinol, an FDA-approved drug that inhibits xanthine oxidase and xanthine dehydrogenase, has been used in clinic for the treatment of gout [36]. Recent studies reveal allopurinol suppresses the expression of HIF-1α in cancer cells and HUVECs [37, 57], and also inhibits tumor growth detected by xenograft and PDX models [58]. In this study, we determined that allopurinol significantly attenuated HIF-1α protein and further inhibited the expression of TMTC3. Besides, the combination therapy of allopurinol and cisplatin induced obvious regression in tumors by xenograft tumor mouse model.

In summary, our study revealed the molecular mechanism of TMTC3 in regulating tumor angiogenesis in ESCC. Further investigations unveiled that TMTC3 inhibitor allopurinol might be an attractive candidate for ESCC treatment, especially when combined with cisplatin. These findings identified a novel therapeutic schedule to enhance cisplatin sensitivity and provided a promising direction in clinical treatment for ESCC in the future.

## Conclusions

**Fig. 8:**
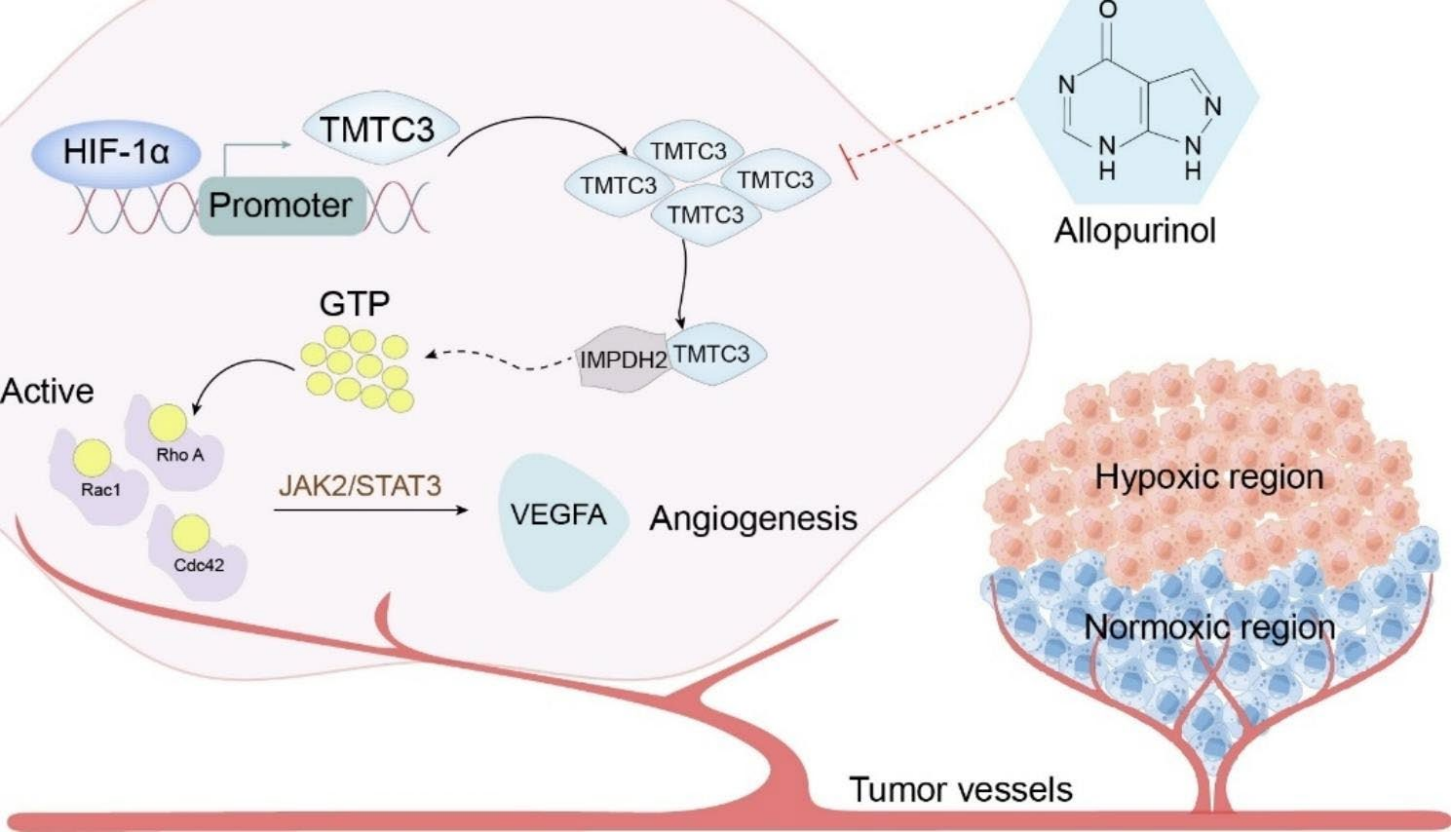
Proposed model of the mechanism through which HIF-1α/TMTC3 controls tumor angiogenesis in ESCC.

In this study, an important role for TMTC3 under hypoxia in promoting ESCC angiogenesis was clarified. The molecular mechanisms suggested that TMTC3 facilitated tumor angiogenesis by regulating Rho GTPase/STAT3/VEGFA pathway mediated by interacting with IMPDH2 Bateman domain. Allopurinol was identified by high-throughput screening of FDA-drugs to reduce TMTC3 expression and enhance cisplatin sensitivity in ESCC (Fig. 8). These findings shed light on new molecular mechanism of TMTC3 in promoting tumor angiogenesis, and TMTC3 might be a potential target and proposed a promising strategy for the treatment of ESCC.

### Electronic supplementary material

Below is the link to the electronic supplementary material.

Supplementary Material 1

Supplementary Material 2

## Data Availability

The data that support the findings of this study are available in the supplementary material.

## Abbreviations

ATF4: activating transcription factor 4
CAM: chorioallantoic membrane
ChIP: chromatin immunoprecipitation
CMC-Na: sodium carboxymethyl cellulose
CoCl_2_: cobalt chloride
EC: esophageal cancer
EGF: epithelial growth factor
ER: endoplasmic reticulum
ESCC: esophageal squamous cell carcinoma
FBS: fetal bovine serum
FGF: fibroblast growth factor
GRP78: glucose-regulated protein, 78kDa
GTPase: guanosine triphosphatase
HIF-1α: hypoxia inducible factor 1 alpha
HUVEC: human umbilical vein endothelial cell
IC50: half-maximal inhibitory concentration
IMPDH2: inosine-5’-monophosphate dehydrogenase 2
PDGF: platelet-derived growth factor
PERK: PRKR-Like endoplasmic reticulum kinase
ROS: reactive oxygen species
STAT3: signal transducer and activator of transcription 3
TGF-β: transforming growth factor beta
TME: tumor microenvironment
TMTC3: transmembrane and tetratricopeptide repeat (TPR) repeat-containing protein 3
UPR: unfolded protein response
VEGF: vascular endothelial growth factor
XBP1: X-box binding protein 1
