# Supplementary material for: Hypoxia-induced TMTC3 expression in esophageal squamous cell carcinoma potentiates tumor angiogenesis through Rho GTPase/STAT3/VEGFA pathway
Source: J Exp Clin Cancer Res. 2023 Sep 26;42:249. doi: 10.1186/s13046-023-02821-y (PMC10521530; doi:10.1186/s13046-023-02821-y)
Supplement: Supplementary file 2 — Supplementary Material 2 [file 13046_2023_2821_MOESM2_ESM.docx]

**Hypoxia-induced TMTC3 expression in esophageal squamous cell carcinoma potentiates tumor angiogenesis through Rho GTPase/STAT3/VEGFA pathway**

Hongyu Yuan^1, †^, Zitong zhao^2, †^, Jing Xu^3^, Ruiping Zhang^3^, Liying Ma^2^, Jing Han^4^, Weihong Zhao^5^, Mingzhou Guo^1, *^, Yongmei Song^2, *^

**Affiliations:**

^1^Department of Gastroenterology & Hepatology, The First Medical Center, Chinese PLA General Hospital, 28 Fuxing Road, Beijing, 100853, China.

^2^State Key Laboratory of Molecular Oncology, National Cancer Center/National Clinical Research Center for Cancer/Cancer Hospital, Chinese Academy of Medical Sciences and Peking Union Medical College, Beijing 100021, China.

^3^State Key Laboratory of Bioactive Substance and Function of Natural Medicines, Institute of Materia Medica, Chinese Academy of Medical Sciences and Peking Union Medical College, 100050 Beijing, China.

^4^Department of Medical Oncology, Hebei Medical University Fourth Affiliated Hospital and Hebei Provincial Tumor Hospital, Shijiazhuang, Hebei 050000, China.

^5^Medical Department, Chinese PLA General Hospital, 28 Fuxing Road, Beijing, 100853, China.

Correspondence to: *To whom correspondence should be addressed. Yongmei Song, State Key Laboratory of Molecular Oncology, National Cancer Center/National Clinical Research Center for Cancer /Cancer Hospital, Chinese Academy of Medical Sciences and Peking Union Medical College, Beijing, China. Tel: 86-010-8778-8422, Fax: 86-10-6771-5058, Email: symlh2006@163.com, songym@cicams.ac.cn. Mingzhou Guo, Department of Gastroenterology & Hepatology, The First Medical Center, Chinese PLA General Hospital, Beijing, China. Email: mzguo@hotmail.com.

^†^These authors contributed equally to this work.

**Supplementary Tables**

**Supplementary table 1** The siRNA sequences for IMPDH2 and primers for qPCR.

The siRNA sequences for IMPDH2.

| Gene | Sense (5’-3’) | Antisense (5’-3’) |
| --- | --- | --- |
| IMPDH2 |  |  |
| Si-1 | GGACAGACCUGAAGAAGAATT | UUCUUCUUCAGGUCUGUCCTT |
| Si-2 | GCCAGGACAUUGGUGCCAATT | UUGGCACCAAUGUCCUGGCTT |

Primer sets for qPCR analysis.

| Gene | Forward | Reverse |
| --- | --- | --- |
| TMTC3 | TGGTTACTGCCTGCTATTGGA | GTGGCTTCTCTCCTCAGACA |
| β-actin | CTCCATCCTGGCCTCGCTGT | GCTGTCACCTTCACCGTTCC |
| 18s | CAGCCACCCGAGATTGAGCA | TAGTAGCGACGGGCGGTGT |
| IMPDH2 | CTCCCTGGGTACATCGACTT | GCCTCTGTGACTGTGTCCAT |
| HIF-1A | AGCTTGCTCATCAGTTGCCA | TCCAAATCACCAGCATCCAGA |

Primer sets for ChIP-qPCR analysis.

| Gene | Forward | Reverse |
| --- | --- | --- |
| TMTC3-bs1/2 | AGTATTAGGGACCCGAGGCTG | CTTGGTGGTTGCGGTAGTGAG |
| TMTC3-bs3 | CCTCTCACTACCGCAACCACC | TAGGCTCTCCTGGCATCC |
| TMTC3-bs4/5 | CGACACCATCCCCAACTCC | TGCAGAAGCCTCAGGCAGA |

**Supplementary table 2** The high throughput screening data for TMTC3 inhibitor.

**Supplementary figures**


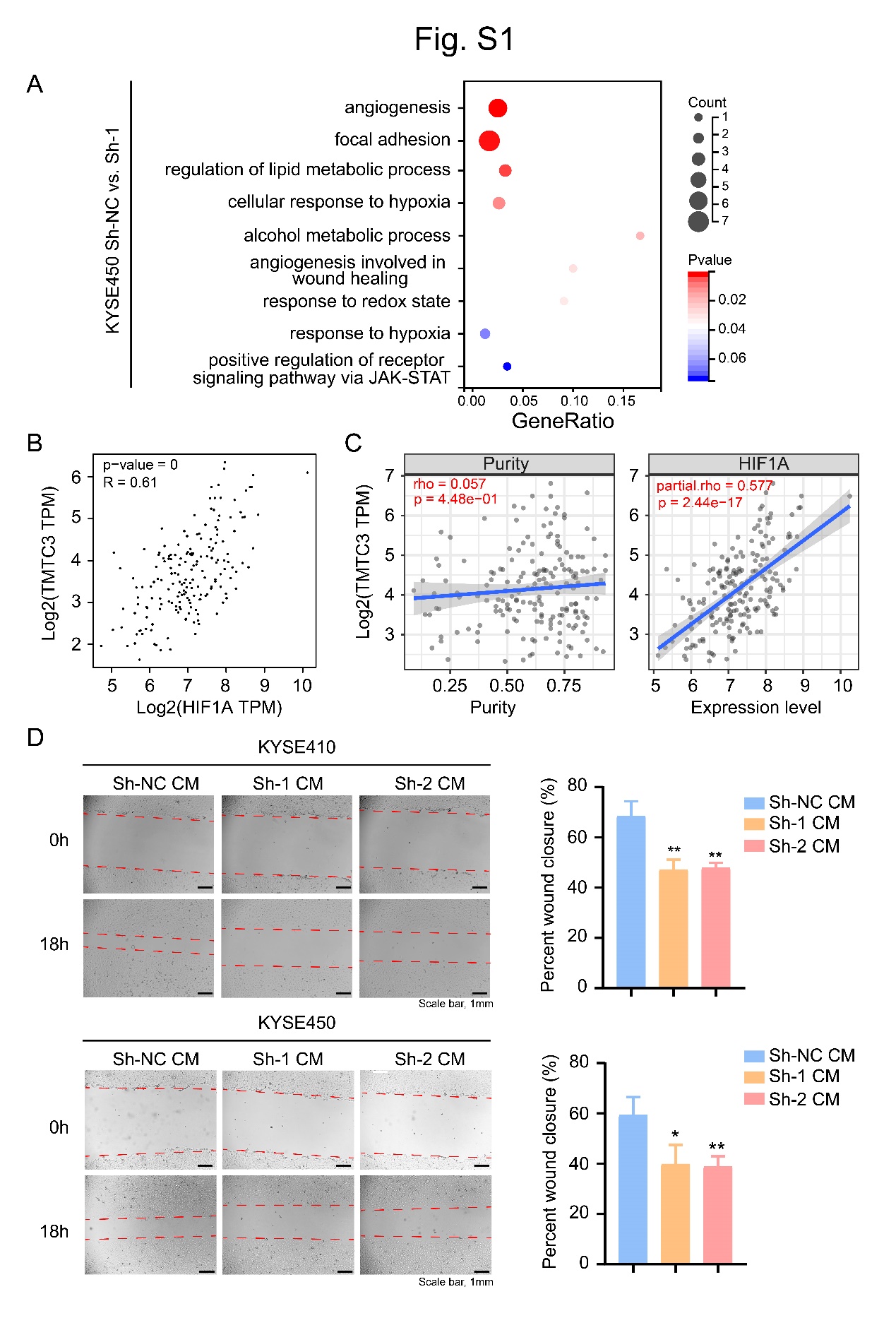


**Supplementary Fig. S1 The role of TMTC3 under hypoxia in ESCC.**

A. The enrichment pathway analysis in TMTC3 knockdown cells. B and C. The correlation between TMTC3 and HIF-1α in esophageal cancer from GEPIA2 (B) and TIMER2.0 (C) database, respectively. D. HUVECs wound closure after incubation with conditioned medium from TMTC3 knockdown cells or negative control cells. All data are expressed as the mean ± SD. *, p < 0.05, **, p < 0.01. n = 3.


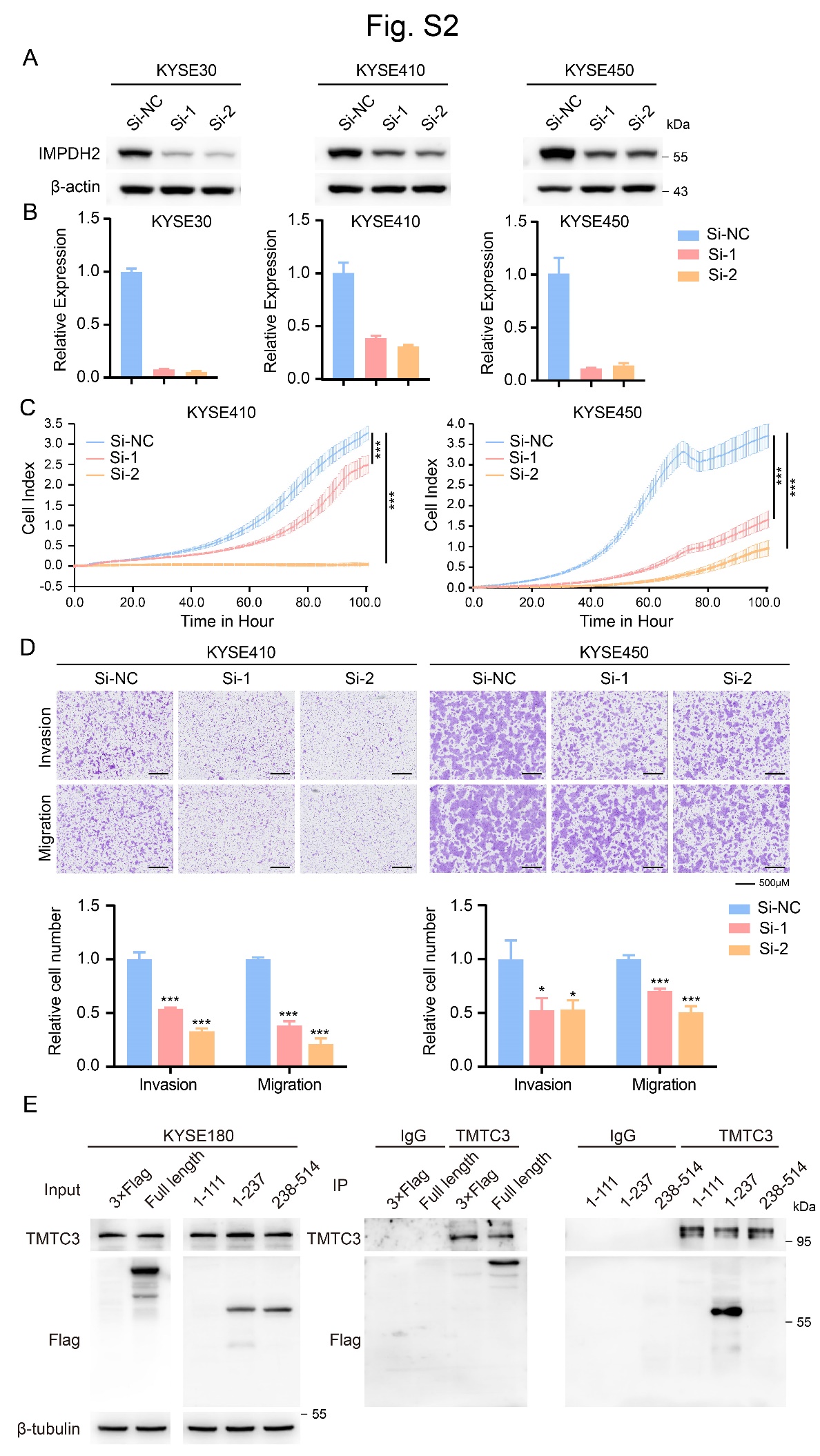


**Supplementary Fig. S2 Knockdown of IMPDH2 reduced the malignant phenotype in ESCC.**

A and B. The knockdown efficacy of IMPDH2 at the protein (A) and mRNA level (B) in three ESCC cell lines. C. Cell proliferation in IMPDH2 inhibition cells via RTCA assay. D. The representative images (up) and quantification results (down) of the transwell assay in IMPDH2 knockdown cells. E. Indicated Flag-tagged IMPDH2 mutants were transfected into KYSE180 cells, followed by immunoprecipitation with anti-TMTC3 antibody and immunoblotting with anti-Flag antibody. All data are expressed as the mean ± SD. *, p <  0.05. **, p <  0.01. ***, p <  0.001. n = 3.


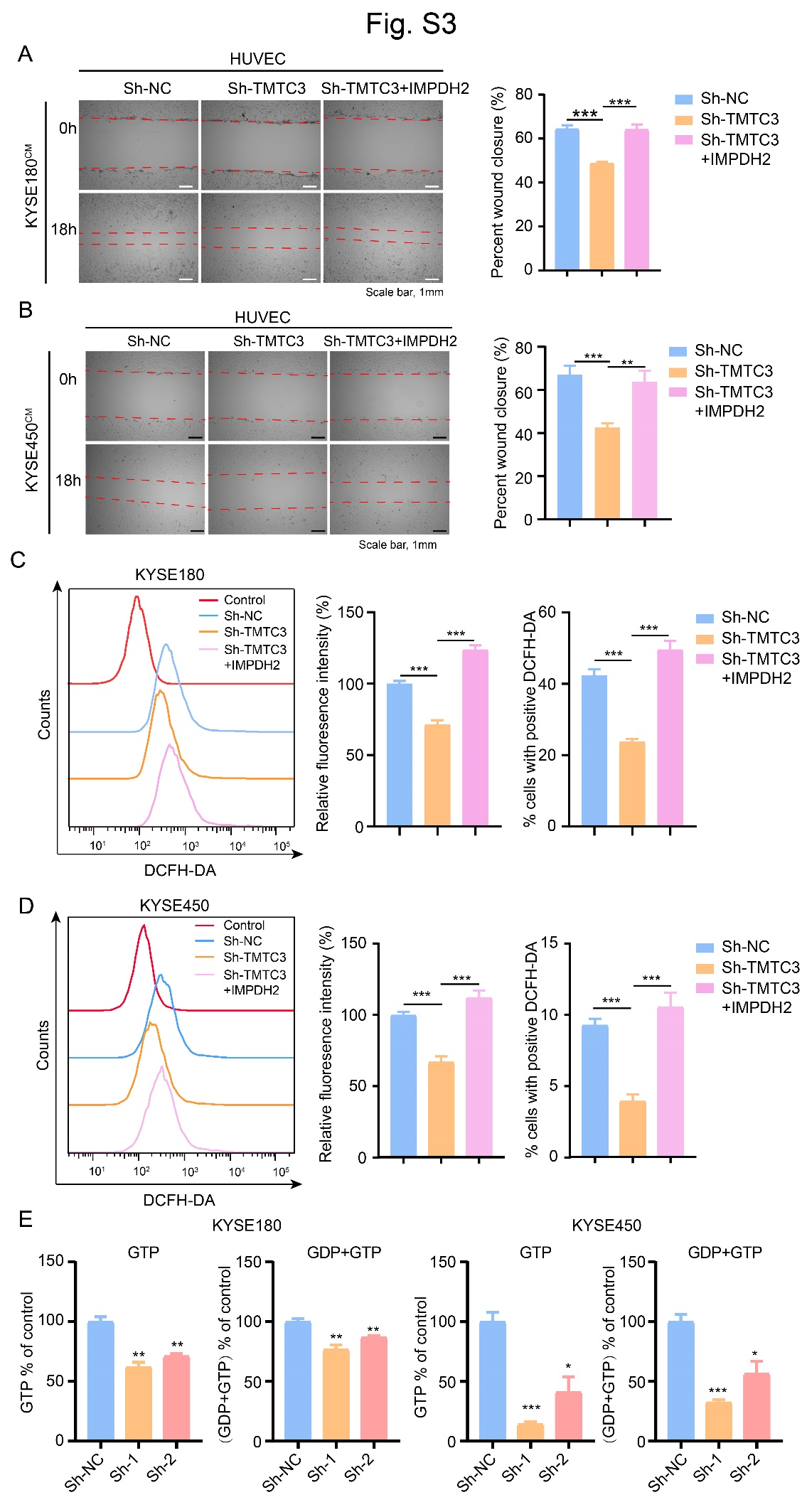


**Supplementary Fig. S3 IMPDH2 overexpression blunted the migration of HUVECs and ROS production caused by TMTC3 knockdown.**

A and B. HUVECs wound closure after incubation with conditioned medium as indicated on the top in KYSE180 (A) and KYSE450 (B) cells. The percentage of wound closure was analyzed by Image J software. C and D. ROS accumulation was analyzed by flow cytometry with DCFH-DA probe staining in KYSE180 (C) and KYSE450 (D) cells. The positive cells and fluorescence intensity of cells were calculated. E. The GTP and GDP levels in TMTC3 knockdown cells were detected by mass spectrometry. All data are expressed as the mean ± SD. *, p < 0.05; **, p < 0.01; ***, p < 0.001. n = 3.


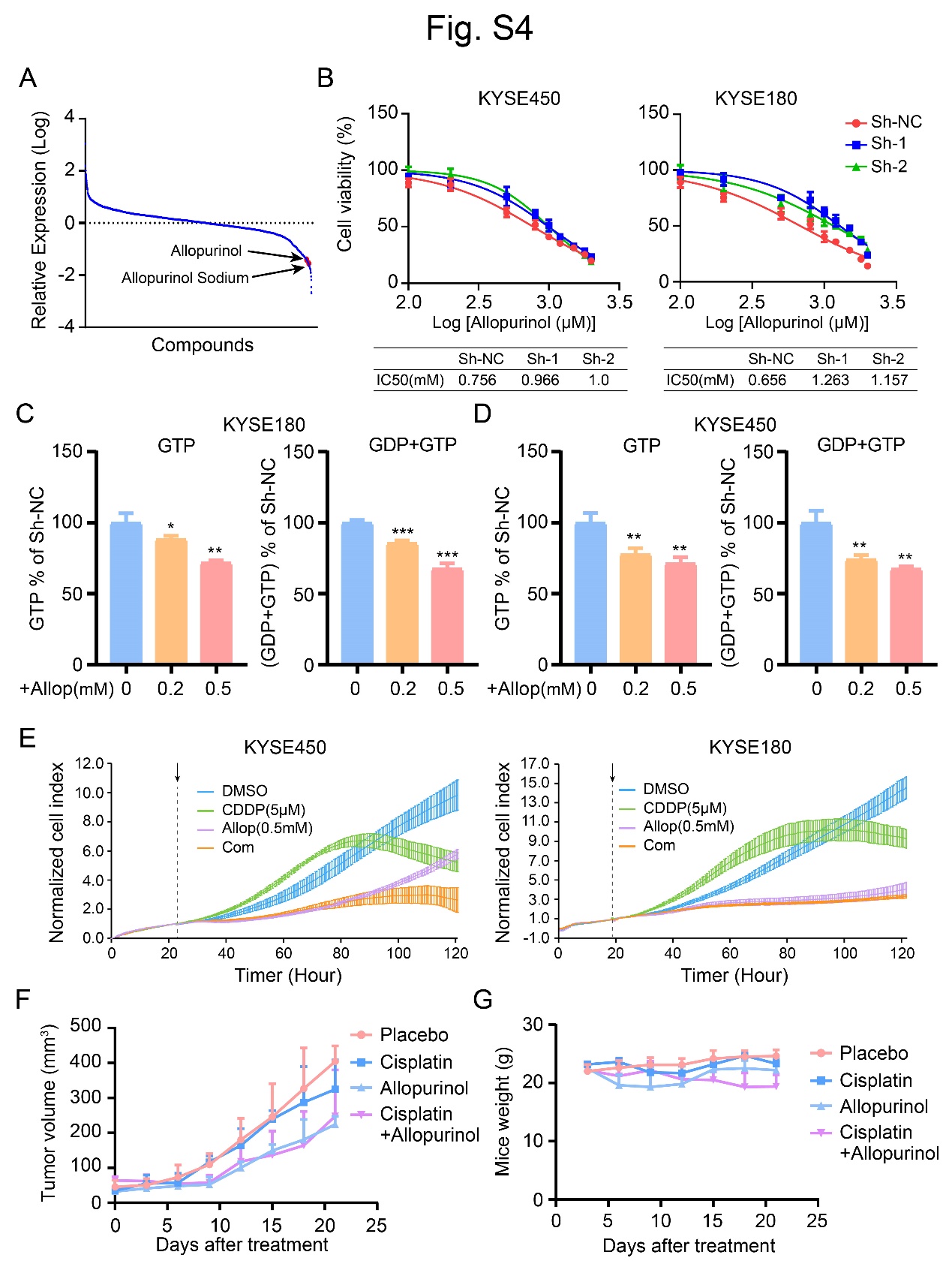


**Supplementary Fig. S4 TMTC3 inhibitor allopurinol inhibited GTP levels and enhanced sensitivity to cisplatin.**

A. High throughput screening of FDA-approved compounds against TMTC3. B. Growth inhibition curves of TMTC3 knockdown cells or control cells upon treatment with different doses of allopurinol. IC50 was measured by SPSS22. C and D. The GTP and GDP levels in KYSE180 (C) and KYSE450 (D) cells upon treatment with allopurinol. n = 3. E. The cell viability of KYSE450 and KYSE180 cells after treatment with allopurinol, or cisplatin, or combination. n = 3. F. Tumor volume curves for each group during the whole therapy. n = 5. G. The mice weight curves for each group during in the therapeutic course. n = 5. All data are expressed as the mean ± SD. *, p < 0.05; **, p < 0.01; ***, p < 0.001.
